# Risk Factors for Chronic and Recurrent Otitis Media–A Meta-Analysis

**DOI:** 10.1371/journal.pone.0086397

**Published:** 2014-01-23

**Authors:** Yan Zhang, Min Xu, Jin Zhang, Lingxia Zeng, Yanfei Wang, Qing Yin Zheng

**Affiliations:** 1 Department of Otorhinolaryngology-HNS, Second Hospital, Xi’an Jiaotong University School of Medicine, Xi’an, China; 2 Departments of Otolaryngology-HNS and Genetics, and the Case Comprehensive Cancer Center, Case Western Reserve University, Cleveland, Ohio, United States of America; 3 Transformative Otology and Neuroscience Center, Binzhou Medical University, Yantai, Shandong, China; Columbia University, College of Physicians and Surgeons, United States of America

## Abstract

Risk factors associated with chronic otitis media (COM) and recurrent otitis media (ROM) have been investigated in previous studies. The objective of this study was to integrate the findings and determine the possible risk factors for COM/ROM based on our meta-analysis. A comprehensive search of electronic bibliographic databases (PubMed, Embase, CNKI and Wanfang database) from 1964 to Dec 2012, as well as a manual search of references of articles, was performed. A total of 2971 articles were searched, and 198 full-text articles were assessed for eligibility; 24 studies were eligible for this meta-analysis. Regarding risk factors for COM/ROM, there were two to nine different studies from which the odds ratios (ORs) could be pooled. The presence of allergy or atopy increased the risk of COM/ROM (OR, 1.36; 95% CI, 1.13–1.64; *P* = 0.001). An upper respiratory tract infection (URTI) significantly increased the risk of COM/ROM (OR, 6.59; 95% CI, 3.13–13.89; *P*<0.00001). Snoring appeared to be a significant risk factor for COM/ROM (OR, 1.96; 95% CI, 1.78–2.16; *P*<0.00001). A patient history of acute otitis media (AOM)/ROM increased the risk of COM/ROM (OR, 11.13; 95% CI, 1.06–116.44; *P* = 0.04). Passive smoke significantly increased the risk of COM/ROM (OR, 1.39; 95% CI, 1.02–1.89 *P* = 0.04). Low social status appeared to be a risk factor for COM/ROM (OR, 3.82; 95% CI, 1.11–13.15; *P* = 0.03). Our meta-analysis identified reliable conclusions that allergy/atopy, URTI, snoring, previous history of AOM/ROM, Second-hand smoke and low social status are important risk factors for COM/ROM. Other unidentified risk factors need to be identified in further studies with critical criteria.

## Introduction

Chronic otitis media (COM) and recurrent otitis media (ROM) are two of the most common infectious diseases worldwide. COM and ROM affect diverse cultural and racial groups that are distributed in both developing and industrialized countries. A cross-sectional study conducted in nine countries over three continents revealed that disease prevalence is significant enough to be considered for clinical practice [1]. COM/ROM can cause hearing impairment and speech delay. COM can cause both intracranial and extracranial complications [2]. Effective treatment of the diseases depends on a thorough understanding of the risk factors.

Risk factors associated significantly with COM/ROM include ethnicity [3]–[5], genetic factors [6], gender [7], day-care center attendance [8], breast-feeding [9], and allergy/atopy [10] etc. as reported in previous studies. However, many of the reported studies were difficult to compare because they lacked clear case definitions, standard diagnostic criteria or control groups to evaluate the potential study biases. We conducted a meta-analysis of all available published data and qualified studies that investigated the potential risk factors for COM/ROM to clarify and propose possible means of treatment of the disease.

## Materials and Methods

### Study Identification

**Figure 1:**
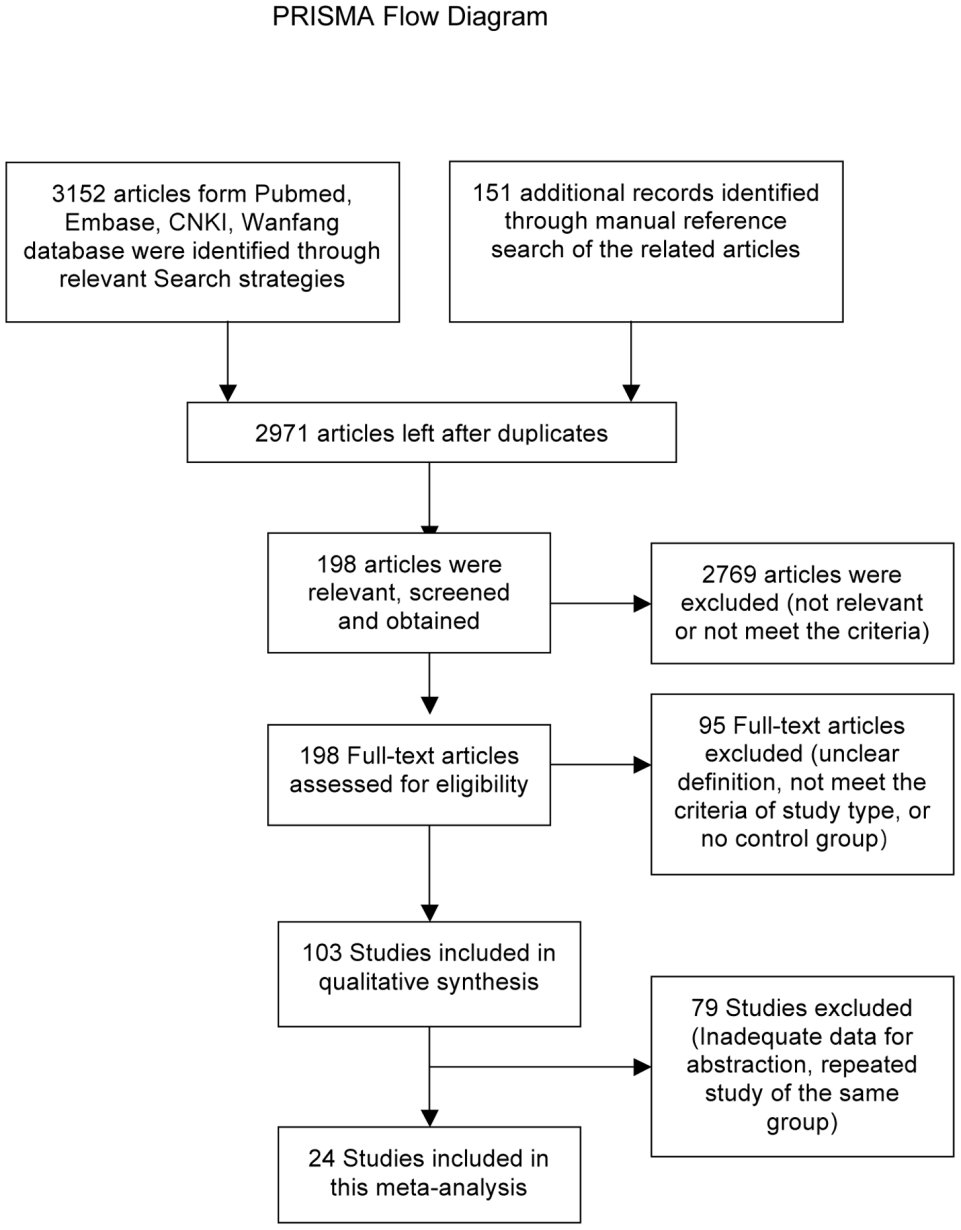
PRISMA Flow Diagram.

A literature search was conducted manually according to the search strategy (Text S1) to evaluate the risk factors for COM/ROM. We searched for the articles published in Pubmed, Embase, WanFang data (http://www.wanfangdata.com.cn/) and China National Knowledge infrastructure (CNKI) database (http://dlib.edu.cnki.net/kns50/). Articles from 1964 to Dec 2012 were included in the search. The search was limited to humans and performed with no language restrictions. Reference lists of the relevant original and reviewed articles were evaluated to identify additional studies. We used controlled vocabularies (Explosion mapped searches of MeSH terms or Emtree thesaurus terms) and text words for chronic otitis media, recurrent otitis media, middle ear cholesteatoma, and mastoiditis. Concepts related to “Otitis media” with the subheadings of congenital, epidemiology, genetics, immunology, microbiology and virology for all Mesh terms in PubMed were reviewed. Areas of focus that were chosen for otitis media in Embase were genetics, immunology and hematology, microbiology, otorhinolaryngology, pediatrics and public health. Furthermore, terms indicating risks, such as “risk factors”, “probability”, “odds ratio”, “risk assessment”, “causality”, “epidemiologic factors”, “epidemiology”, “epidemiologic studies”, “multivariate analysis”, “logistic models” and their entry terms were also included (see Text S1). Overall, 2547 papers were retrieved from Pubmed, 479 papers were retrieved from Embase, 116 papers were retrieved from CNKI and 10 were retrieved from Wanfang. A total of 151 additional records were retrieved from the manual reference search of the related articles. The workflow of this study follows guidelines by the Preferred Reporting Items for Systematic Reviews and Meta-analyses (PRISMA) Statement [11](**Figure 1**).

### Definition of COM/ROM

The diagnosis criteria of COM/ROM was described in individual studies, which included case history, physical examination and other examinations such as tympanogram, microscopic otoscopy or tympanostomy tube insertions (**Table 1**). The abbreviation COM includes the types of chronic suppurative otitis media (CSOM) and chronic otitis media with effusion (COME). Chronic otitis media with cholesteatoma was not excluded from the COM definition, although no study involving that type was eligible for our meta-analysis.

**Table 1:** Characteristics of included studies.

First author	Year of publication	Risk factor	Type of otits meida	Study type	Age, years of participants	Study duration	Number of cases	Number of controls	Total Sample Size	Study Location	Ethnic Group	Diagnostic criteria of COM/ROM
Stahlberg, M. R. [37]	1986	Day-care center attendance, Passive smoke, Low socioeconomic status	ROM	Case-control	10–44 months in case group, 14–38 months in control group	March, 1983–Feb, 1984	115	222	337	Turku, Finland	Inhabitants in Turku, Finland	Three or more episodes of OME
Daly, K. [8]	1988	Sex, Day-care center attendance, White people, Allergy, Family history of OM	COME	Case-control	10 months - 8 years of age	Jan, 1982-Sep, 1984	177	182	359	Minnesota, USA	White people and others unidentified population	MEE persisted in one or both ears at the 3- and 6 week visits, or AOM without resolution of MEE during the 6 weeks
Fliss, D. M. [21]	1991	History of AOM/ROM, Day-care center attendance, Larger families and more siblings, Sex, Allergy, Sinusitis and recurrent URTI, Breast feeding, Passive smoke	CSOM without Cholesteotoma	Case-control	2–15 years of age	Jan, 1987-April, 1990	88	76	164	Southern Israel	Jewish population	Continuous otorrhea ≥2 months
Kalm, O. [27]	1994	HLA frequency	CSOM	Follow-up	Mean age 16.4	Follow up 11.1 years	40	1701 for HLA-A and B 438 for HLA-C 102 for HLA-DR	1741	Sweden	No comment	Chronic or recurrent mucous middle ear secretion persisting for at least 6 years.
Kvaerner, K. J. [29]	1996	Birth weight, Gestational age	ROM	Case-control	Before age 7	Baby born between 1967–1974	519	5345	5864	Norway	Norwegian twin pairs	Recurrent ear infections
Ilicali, O. C. [24]	1999	Passive smoke, Sex	ROM	Follow-up	3–7 years of age	May 1^st^, 1995– Nov 30^th^, 1996	166	166	332	Istanbul, Turkey	Patients from Istanbul School of Medicine	Extensive OM bilateral for at least 3 months or 6 months unilateral. ≥3 episodes of AOM during previous 6 months or minimum 4 episodes during previous 1 year.
Juntti, H. [26]	1999	Cow's milk allergy	ROM	Case control	9–11 years of age. Mean age = 10.5±0.6 years	1986–1987	56	204	260	Finland	Local residents	15 episodes of OM in 10 years
Engel, J. [19]	2001	Sex, Gestational age, Birth weight	COME	Prospective cohort	2 years of age	2- year follow-up	43	40	83	Netherland	Newborns from Maastricht University Hospital	Otoscopy and tympanoetry examination to assessed combined with MOMES diagnostic-algorithm
Ilicali, O. C. [23]	2001	Passive smoke	ROM	Follow-up	3–8 years of age	Oct, 1996-Apr, 1998	114	40	154	Istanbul, Turkey	Local residents	OME persisted for ≥3 months bilateral or 6 months unilateral. ≥3 episodes of RAOM during previous6 months or ≥4 episodes during the previous year.
Ramet, M. [33]	2001	Surfactant protein- A frequencies	ROM	Case-control	1–10 years of age, mean age = 8.4±5.2	No comment	147	228	375	Finland	Local patients and residents	At least 5 episodes of AOM
Daly, K. A. [17]	2004	Support for linkage at chromosomes 10q and 19q, Day-care center attendance, Exclusively formula fed, Passive smoke	COME/ROM	Retrospective cohort	Family members, age not mentioned	1992–2001	371	245	616	Minnesota,USA	Families recruited from University of Minnesota	Tympanostomy tube surgery for COME/ROM
Keles, B. [28]	2004	Pharyngeal reflux, Gastroesophageal reflux	COME	Prospective cohort	3–7 years, mean age = 6±3.1	No comment	25	12	37	Konya, Turkey	No comment	COME >3 months
Engel, J. A. [20]	2005	Breast feeding, Day-care center attendance, Family history of OM, Passive smoke, Snoring, URTI, Mother's smoking during pregnancy, Medication use during pregnancy	ROM	Prospective cohort	2.1–7.5 years of age	Dec, 1999- Aug, 2003	73	17	90	Nijmegen and Winterswijk, Netherlands	No comment	MEE at least for 3 months
Chantry, C. J. [16]	2006	Breast feeding	ROM	Prospective cohort	6–72 months of age	1988–1994	88	271	359	USA	White, black, Mexican American	>3 episodes of OM
Gozal, D. [22]	2008	Snoring, African American, Chronic nasal obstruction, Allergy, Passive smoke	ROM	Retrospective corhort	5–7 years of age	1999–2004	5074	11247	16321	Louisville, USA	African American and other unclassified ethnic groups	History of ROM and insertion of tympanostomy tubes
Lasisi, A. O. [30]	2009	Serum retinol level	CSOM	Follow-up	6 months–7 years, mean age = 7.8 years	No comment	116	52	168	Ibadan, Nigeria	No comment	Persistence of otorrhoea ≥3 months
Lasisi, A. O. [31]	2007	URTI, Indoor- cooking, Allergy, Low social status group, Passive smoke, Breast- feeding, Day-care center attendance	COME	Case-Control	30 days-14 years of age	No comment	189	100	289	Nigeria	No comment	≥3 episodes of OM in 1 year
Schejbel, L [36]	2009	Properdin deficiency	ROM	Retrospective cohort	All age from three generations of a family	No comment	4	21	25	Denmark	Indian	Several episodes of OM
Bakhshaee, M. [15]	2011	Allergy	CSOM	Prospective cohort	10–50 years, mean age = 30 years)	No comment	68	184	252	Mashad, Iran	No comment	CSOM diagnosed for at least 1 year
Elemraid, M. A. [18]	2011	Nutritional factors	CSOM	Case-control study	0.6–15 years (mean = 6.0) in case group 0.9–15 years (mean = 8.2) in control group	March to May 2007	75	74	149	Sana’a, Yemen	Local children	Diagnosis of CSOM and history of persistent discharging ear(s) for at least 2 weeks
Jensen, R. G. [25]	2011	Sex, Ethnicity, Low education of mother, Family history of COM, Breast feeding	CSOM	Follow-up	11–15 years	1996–2008	45	191	236	Nuuk and Sisimiut, Greenland	Inuit, Danish, Mixed	≥2 weeks of otorrhea for ≥3 months
Nelson, H. M. [32]	2011	Overweight in toddlers	ROM	Prospective cohort	1 month- 27 months. Mean age = 24.1 months	1991–1996	203	227	430	Minneapolis, USA	Local toddlers	ROM treated with tympanostomy tubes
Sale, M. M. [35]	2011	Day-care center attendance, Breast feeding, Allergy	COME/ROM	Case-Control	Mean age = 5.9 in case group. 5.4 in control group	Oct, 1996 - Apr, 1998	380	238	618	Istanbul, Turkey	Local residents	OME or ROM treated with ventilation tubes

Abbreviation: OM: Otitis media. OME: Otitis media with effusion. URTI: upper respiratory tract infection CSOM: Chronic suppurative otitis media. COME: Chronic otitis media with effusion. MEE: Middle ear effusion. ROM: Recurrent otitis media. RAOM: Recurrent acute otitis media MOMES: Maastricht Otitis Media with Effusion Study. TM: Tympanic membrane.

### Study Selection and Quality

We included the study of the prospective cohort, case-cohort, and nested case-control design, case control or nested case-control, retrospective case-control, and cross-sectional studies. The publications included were required to meet the following criteria:

1. Inclusion of human subjects
2. Clear definitions of COM/ROM and estimation of the association of the relative risks (hazard ration, risk factors) of COM/ROM;
3. The numbers for both controls and COM/ROM cases;
4. Sufficient data are to determine the odds ratio (OR) with 95% confidence intervals (CIs).

We excluded descriptive studies, case reports, case series, reviews, letters, commentaries, and studies on the pathogenesis and treatment of COM/ROM. We excluded repeated reports with a small number of participants and these data were included in large studies mentioned above. We excluded the studies of recurrent acute otitis media, congenital cholesteatoma and unclassified OM. Inclusion discrepancy was resolved in joint discussions by the investigators. We appraised the quality of the studies, focusing on the selection of cohorts and assessment of the outcomes.

### Data Extraction

Two investigators, (Yan Zhang and Jin Zhang) independently extracted and registered the data from the eligible publications. The following data from each article was extracted: author, year of publication, risk factor, type of otitis media, study type, age/years of participants, study duration, number of cases, number of controls, total sample size, study location, ethnic group and diagnostic criteria for COM/ROM. All disagreements were resolved through group discussion.

### Statistical Analysis

The meta-analysis was processed using Review Manager 5.1, version: 5.1.6. We estimated the odds ratios (ORs) and 95% confidence intervals (CIs), and the statistical heterogeneity of the studies was assessed before combining the results. Estimates of the risk factors were pooled using a random effects model [12]. Inconsistency of the studies was quantified by using the I^2^ statistic, which describes heterogeneity across studies. I^2^ values of <25% and >50% reflects low and high heterogeneity, respectively [13]. A sensitivity analysis was performed by calculating the outcomes after a single study was omitted in each turn. Finally, publication bias was assessed by performing funnel plots [14] (see Figure S1).

## Results

### Literature Search and Study Selection

Of the total 2971 relevant references identified, 198 articles were considered potentially relevant. The excluded references that were considered irrelevant included reviews, letters, commentaries, studies on pathogenesis, pathologies, and treatment, and microbiological studies. A total of 103 case control or cohort studies examined the risk factors of COM/ROM, and 79 studies failed to meet the inclusion criteria for the following reasons: unclear definition of COM/ROM, no classification of OM, no control groups, and inadequate data for abstraction. For repeated studies, we retained the one with the larger sample size. Figure 1 shows the selection flow for this meta-analysis; 24 independent studies met all of the inclusion criteria [8], [15]–[38]. The characteristics of the included studies are summarized in **Table 1**.

### Pooled Analysis of Risk Factors

Pooled data from 7 studies indicated the presence of allergy or atopy and increased the risk of COM/ROM (OR, 1.36; 95% CI, 1.13–1.64; *P* = 0.001). A total of four studies investigated the association between upper respiratory tract infection (URTI) and COM/ROM, which includes the presence of cough or rhinorrhea or nasal stuffiness or sore throat or adenoiditis/adenoid hypertrophy. Pooled data from these showed that URTI significantly increased the risk of COM/ROM (OR, 6.59; 95% CI, 3.13–13.89; *P*<0.00001). A total of two studies showed that snoring appeared to be a significant risk factor for COM/ROM (OR, 1.96; 95% CI, 1.78–2.16; *P*<0.00001). Pooled data from two studies revealed that a patient history of AOM/ROM increased the risk of COM/ROM (OR, 11.13; 95% CI, 1.06–116.44; *P* = 0.04); nine studies investigated parental smoking, exposure to smoking at home and other smokers residing in the same household of frequent visitors. Pooled data showed Second-hand smoke, including the conditions above, increased the risk of COM/ROM (OR, 1.39; 95% CI, 1.02–1.89 *P* = 0.04). Pooled data from two studies showed low social status as an increased risk factor of COM/ROM (OR, 3.82; 95% CI, 1.11–13.15; *P* = 0.03).

The factors that were determined to not be significantly associated with increased risk included chronic nasal obstruction (OR, 1.19; 95% CI, 0.84–1.69; *P* = 0.34), male sex (OR, 1.24; 95% CI, 0.99–1.54; *P* = 0.06), attending day-care centers (OR, 1.70; 95% CI, 0.95–3.05; *P* = 0.07), family history of otitis media (OR, 1.40; 95% CI, 0.86–2.28; *P* = 0.18), low education of the mother (OR, 1.68; 95% CI, 0.32–8.68; *P* = 0.54), mother's smoking during pregnancy (OR, 2.34; 95% CI, 0.64–8.54; *P* = 0.20), larger families and more siblings (OR, 1.57; 95% CI, 0.93–2.63; *P* = 0.09). Pooled data revealed that an association between breast-feeding >6 months and COM/ROM was not statistically significant (OR, 0.57; 95% CI, 0.17–1.93; *P* = 0.36), neither was an association between breast feeding (yes/no) and COM/ROM (OR, 0.91; 95% CI, 0.47–1.79; *P* = 0.79). Pooled risk factors for COM/ROM are summarized in **Table 2** and Figure S2.

**Table 2:** Pooled analysis of risk factors.

Risk factor	No. of studies[references]	No. of subjects	OR	95% CI	P value	I^2^ (%)
Allergy/Atopy	7 [8], [15], [21], [22], [26], [31], [35]	18263	1.36	[1.13, 1.64]	0.001	26
Upper respiratory tract infections	4 [20], [21], [31], [38]	865	6.59	[3.13, 13.89]	<0.00001	65
Chronic nasal obstruction	2 [22], [31]	16610	1.19	[0.84, 1.69]	0.34	54
Snoring	2 [20], [22]	16411	1.96	[1.78, 2.16]	<0.00001	0
Sex (male)	6 [8], [19], [21], [24], [25], [38]	1435	1.24	[0.99, 1.54]	0.06	0
Attending day-care centers	7 [8], [17], [20], [21], [31], [35], [37]	2454	1.70	[0.95, 3.05]	0.07	89
Family history of otitis media	4 [8], [19], [25], [38]	1166	1.40	[0.86, 2.28]	0.18	52
Patient history of AOM/ROM	2 [21], [38]	425	11.13	[1.06,116.44]	0.04	94
Passive Smoke	9[17], [20]–[24], [31], [37], [38]	18876	1.39	[1.02, 1.89]	0.04	80
Low social status group	2 [31], [37]	600	3.82	[1.11, 13.15]	0.03	82
Low education level of mother	2 [25], [38]	495	1.68	[0.32, 8.68]	0.54	90
Mother's smoking during pregnancy	2 [20], [24]	422	2.34	[0.64, 8.54]	0.20	70
Larger families and more siblings	2 [21], [[38]]	425	1.57	[0.93, 2.63]	0.09	5
Breast feeding >6 months	2 [16], [25]	912	0.57	[0.17, 1.93]	0.36	88
Breast feeding (yes/no)	3 [17], [21], [35]	1363	0.91	[0.47, 1.79]	0.79	86

OR: Odds ratio. 95% CI: 95% confidential intervals. I^2^ describes heterogeneity across studies.

Other risk factor investigations for COM/ROM included in our eligible studies included HLA frequencies [27], nutritional factors [18], medication use during pregnancy [20], ethnicities of Greenland [25], White [8], African American [22], properdin deficiency [36], indoor cooking [31], pharyngeal reflux [28], overweight status [32], older siblings [38], dietary history [18], serum retinol [30], genome scan for loci of 10q and 19q [17], and?Surfactant protein-A gene locus [33]. Unfortunately, only one research group reported each risk factor above, which made the data unavailable. There was an association between gestational age and COM/ROM from two research groups [19], [29], but birth weight and COM/ROM from these groups applied different criteria and made it impossible to combine the data.

## Discussion

COM/ROM is a disease with different possible etiologies. Using a meta-analysis design applying strict diagnostic and inclusion criteria, we performed a reliable study to investigate the risk factors associated with the disease. This study is to the best of our knowledge the first meta-analysis investigating the risk factors for COM/ROM. There are two published studies on the risk factors and etiology of AOM [39], [40].

Allergy or atopy is a significant risk factor for COM/ROM. Indoor allergens and respiratory allergies such as allergic rhinitis contribute to the onset of COM/ROM. The prevalence of atopic conditions, including allergic rhinitis in patients with COM/ROM ranges from 24% to 89% [41]. New evidence from cellular biology and immunology explained allergy as a cause for Eustachian tube (ET) obstruction [42]. People with allergic or atopic conditions are more likely to suffer from COM/ROM.

Upper respiratory tract infection (URTI), which includes the presence of cough or rhinorrhea or sore throat, was indicated as a significant prognostic factor for COM/ROM. Studies support that the mucosal condition of ET could be affected by URTI [43]. A preceding or concurrent viral URTI, as well as a poly-microbial disease is considered one of the risk factors for the onset of OM. Viral URTI promotes the replication of the bacterial infection and increases inflammation in the nasopharynx and ET [44].

Snoring, defined as the presence of loud snoring at least three times per week, is a common symptom in children and is highly prevalent in children [45]. Eligible studies in this meta-analysis suggested that the risk for COM/ROM appeared to be related to the presence of snoring. Snoring is pathophysiologically determined by the size of the upper airway lymphadeniod tissue size [46]. The mechanism underlying snoring and COM/ROM appears to be increasing upper airway resistance as well as Eustachian tube dysfunction [22]. Early evaluation and intervention in children with loud snoring may prevent them from developing middle ear disease.

Previous history of AOM/ROM was studied as a predictive factor for COM/ROM. Subjects who experience episodes of AOM/ROM have an increased risk of developing chronic and recurrent middle ear infections.

Second-hand smoke has been reported to be associated with increased prevalence of middle ear disease [47]. In the meta-analysis of risk factors for acute otitis media, it was concluded that parental smoking increased the onset of acute middle ear infectious disease in children [39]. Our study drew the same conclusion about Second-hand smoking as a remarkable causative factor that contributes to the morbidity of COM/ROM. Several studies suggest that nicotine and other smoking products could make subjects more susceptible to ear infections and enhance the possibility of microorganism invasion to the middle ear. Smoke exposure could impair the mucociliary function of the ET, resulting in blockage of the nasopharyngeal airway [48]. Microorganism adherence to the epithelial cell surface and depression of local immune function were both investigated as the pathogenetic mechanism of the onset of middle ear disease caused by Second-hand smoking [49]. Effective methods should be urgently taken to decrease the prevalence of the smoke exposure.

The possibility that COM/ROM is associated with low social status has been debated for a long period of time [50]. Our data from two eligible studies considered the social prestige of professions and occupations, as well as income earnings of the parents. The statistical data revealed that patients with COM/ROM were more often belonged to low socioeconomic conditions than the controls. Various reports concerning this hazard originated from poor housing, environmental and occupational conditions [51], [52].

Sex difference in otitis media risk has been estimated in various studies. Other than a conclusion that the male sex was more likely to suffer from acute otitis media in children [39], our study failed to find any significance in the difference between male and female morbidity of COM/ROM.

Breast-feeding is believed to provide antimicrobial, anti-inflammatory, and immunomodulatory agents that contribute to an optimal immune system [53]. The relative contribution of breast-feeding to preventing middle ear infection otitis media risk has been reported in numerous studies [54]–[56]. It is reported that breast-feeding, even for only 3 months, could decrease the risk for acute otitis media in children [39]. However, patients with COM/ROM did not differ from the control group in this respect in our study. The study for preventative effects of breast-feeding over 6 months failed to find statistical significance within the control group. Even without any breast-feeding, the impact on the incidence of COM/ROM appeared to be unremarkable in our meta-analysis.

Day-care center attendance could increase the risk of children’s exposure to respiratory pathogens. It has been reported to be a significant risk factor for acute respiratory infectious disease in children [39], [52], [57]. However, this was not consistent with some other studies [58]. In this meta-analysis, no association was found between COM/ROM and day-care center attendance.

The causal relationship between other factors, which include chronic nasal obstruction, family history of otitis media, mother's smoking during pregnancy and COM/ROM is not completely established. Association between larger families and more siblings with COM/ROM was not statistically significant.

Genetic predisposition is considered to be an important prognostic factor that could influence the risk of otitis media. Previous candidate gene studies associated a number of immune system genes with otitis media, which included TNF-α, IL-6, IL-10, Tlr4, surfactant, CD14, FcγRIIa, IFNγ, Eya4, p73, MyD88, Fas, E2f4, Plg, Fbxo11, and Evi1 [59]. Other genetic predispositions include HLA frequencies and properdin deficiency. Unfortunately, eligible studies included in our meta-analysis investigated single gene defects in each study, which made it impossible to pool the data and make a conclusion.

Similar to risk factors for genetic predisposition, other risk factors for COM/ROM prevented the data from being pooled in our eligible studies; these include nutritional factors, medication use during pregnancy, ethnicities of Greenland, White, African American, indoor cooking, pharyngeal reflux, and overweight status.

The association between gestational age and COM/ROM from the two groups [19], [29], birth weight and COM/ROM from the same groups applied different criteria and thus made it impossible to combine the data.

We noticed that the risk factors of sex, attending day-care centers, large families and more siblings have p-values of 0.06, 0.07 and 0.09, respectively. With the application of the 0.05 p-value, which is the conventionally used criterion, no significance was found. Using a cut-off of 10% for significance may ameliorate this problem but could increase the risk of drawing a false positive conclusion (type I error) [13], [60]. However, these three risk factors should be at least considered as constituting a strong trend of risk factors for COM/ROM.

In judging the inconsistency of the studies, I^2^ was applied to test heterogeneity. In our studies of no inconsistency (I^2^ = 0) or low heterogeneity (I^2^<25%), using either fixed or random effect models produced identical results and the same direction of effect. The random effect model was the standard approach for the studies of moderate to high I^2^ values. Some analysts might try to reduce the heterogeneity by limiting the meta-analysis to a smaller more homogeneous study group. However, this could probably result in misleading conclusions if not performed with care or may limit the scope of the meta-analysis and essentially eliminates any useful information [61]. The random effect model, which was the available model to incorporate and evaluate sources of heterogeneity [12], was applied in our study. In our meta-analysis, a limited number of included studies confined our attempts to divide the studies into subgroups. Sources of between-study heterogeneity could probably originate from different study designs, sample size in each individual study, incidence rates among unexposed, length of follow-up, and/or study qualities. In our sensitivity analysis, the observed directions and magnitudes of effects weren’t changed significantly after a single study was randomly omitted in each turn.

A full understanding of the etiologic factors for COM/ROM could be beneficial for the treatment and prevention of the disease. Our study evaluates the risk factors by an objective scientific procedure, meta-analysis, to provide precise causal prophylaxis evidence. Meta-analysis is widely used in medical studies of randomized clinical trials, as well as etiologic factors of the disease. The controversy of meta-analysis is in the homogeneity of the studies. Dickersin and others noted that heterogeneity is not all that bad [62], [63]. It improves the generalizability of the meta-analysis results. The pooled estimates of odds ratios are valuable and important indicators for assessing the risk factors of a disease. The heterogeneity of risk factors is carefully estimated, and the results are cautiously interpreted in our study.

## Conclusions

The risk factors for COM/ROM are closely interrelated. Our meta-analysis identified reliable conclusions that allergy/atopy, upper respiratory tract infection, snoring, previous history of AOM/ROM, Second-hand smoke, low social status are important risk factors for COM/ROM. Other unidentified risk factors investigated in single studies need possible repeated studies with critical criteria to be estimated properly. We suggest that the above COM/ROM risk factors be interfered effectively to prevent and decrease the onset of the disease.

## Supporting Information

Figure S1 **Funnel plot**. Symmetric inverted funnel shape indicates unlikely publication bias.
(TIFF)

Figure S2 **Risk factors for COM/ROM.** Pooled odds ratios from eligible studies analyzed in the meta-analysis of risk factors for COM/ROM
(TIFF)

Checklist S1 **PRISMA checklist.**
(DOC)

Text S1 **Search strategy.**
(DOC)
