# Supplementary figures and images for: Risk Factors for Chronic and Recurrent Otitis Media–A Meta-Analysis
Source: PLoS One. 2014 Jan 23;9(1):e86397. doi: 10.1371/journal.pone.0086397 (PMC3900534; doi:10.1371/journal.pone.0086397)

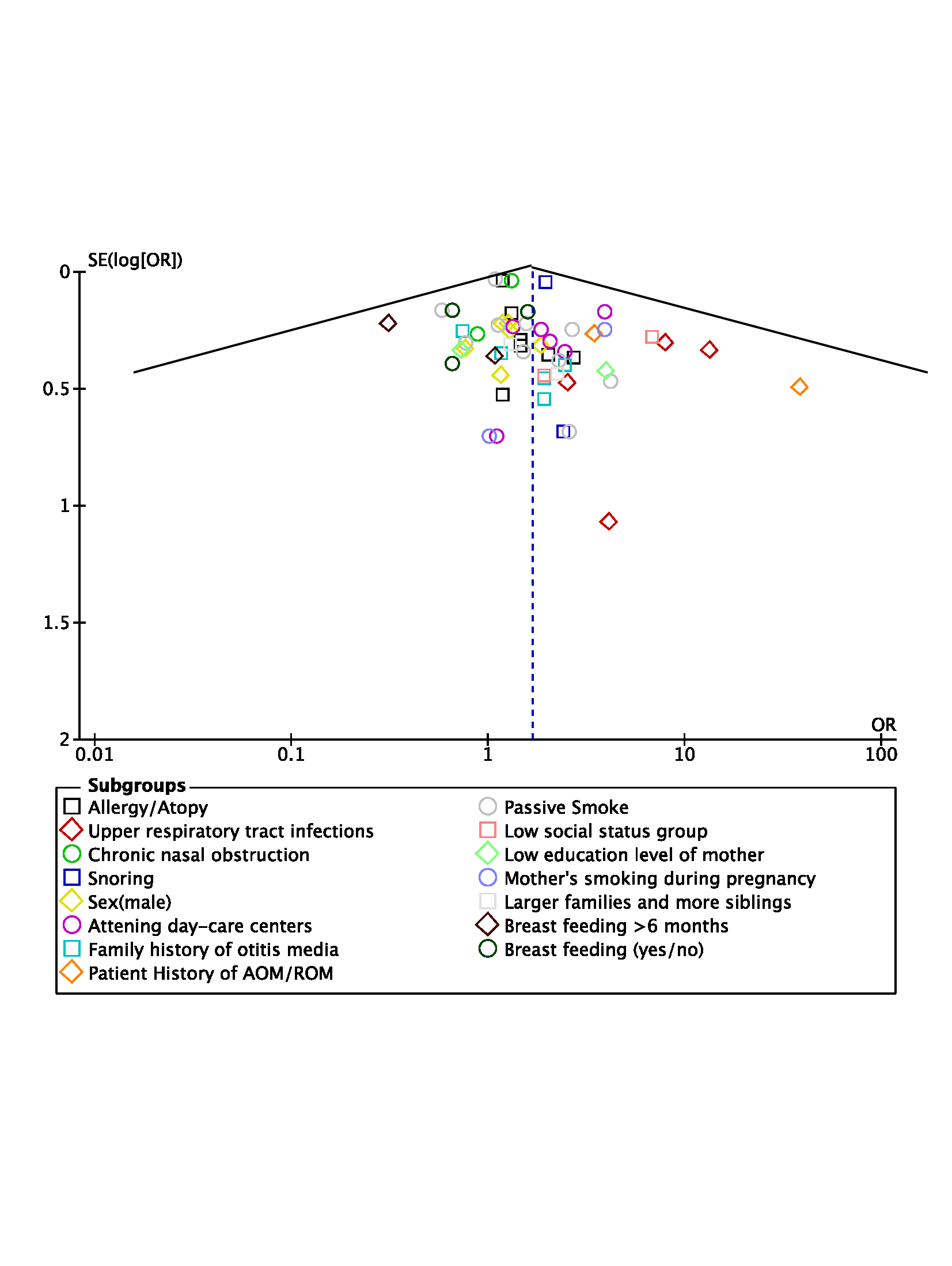

Supplement: Figure S1 — Funnel plot. Symmetric inverted funnel shape indicates unlikely publication bias. (TIFF) [file pone.0086397.s001.tif]
