# Supplementary material for: Risk Factors for Chronic and Recurrent Otitis Media–A Meta-Analysis
Source: PLoS One. 2014 Jan 23;9(1):e86397. doi: 10.1371/journal.pone.0086397 (PMC3900534; doi:10.1371/journal.pone.0086397)
Supplement: Text S1 — Search strategy. (DOC) [file pone.0086397.s004.doc]

**Pubmed Search strategy**

#1 Search "Otitis Media/congenital"[Mesh] OR "Otitis Media/epidemiology"[Mesh] OR "Otitis Media/etiology"[Mesh] OR "Otitis Media/genetics"[Mesh] OR "Otitis Media/immunology"[Mesh] OR "Otitis Media/microbiology"[Mesh] OR "Otitis Media/virology"[Mesh]

#2 Search "Otitis Media, Suppurative/congenital"[Mesh] OR "Otitis Media, Suppurative/epidemiology"[Mesh] OR "Otitis Media, Suppurative/etiology"[Mesh] OR "Otitis Media, Suppurative/genetics"[Mesh] OR "Otitis Media, Suppurative/immunology"[Mesh] OR "Otitis Media, Suppurative/microbiology"[Mesh] OR "Otitis Media, Suppurative/virology"[Mesh]

#3 Search "Otitis Media with Effusion/epidemiology"[Mesh] OR "Otitis Media with Effusion/etiology"[Mesh] OR "Otitis Media with Effusion/genetics"[Mesh] OR "Otitis Media with Effusion/immunology"[Mesh] OR "Otitis Media with Effusion/microbiology"[Mesh] OR "Otitis Media with Effusion/virology"[Mesh]

#4 Search “inflammatory middle ear diseases” [Text Word]

#5 Search "Cholesteatoma, Middle Ear/congenital"[Mesh] OR "Cholesteatoma, Middle Ear/epidemiology"[Mesh] OR "Cholesteatoma, Middle Ear/etiology"[Mesh] OR "Cholesteatoma, Middle Ear/genetics"[Mesh] OR "Cholesteatoma, Middle Ear/immunology"[Mesh] OR "Cholesteatoma, Middle Ear/microbiology"[Mesh] OR "Cholesteatoma, Middle Ear/virology"[Mesh]

# 6 Search "Mastoiditis/epidemiology"[Mesh] OR "Mastoiditis/etiology"[Mesh] OR "Mastoiditis/immunology"[Mesh] OR "Mastoiditis/microbiology"[Mesh] OR "Mastoiditis/virology"[Mesh]

#7 Search #1 OR #2 OR #3 OR # 4 OR #5 OR #6

#8 Search (chronic*[TIAB] OR recurrent*[TIAB])

#9 Search #7 AND #8

# 10 Search #9 NOT “Case Reports” [Publication Type]

# 11 Search #10 AND "Humans"[MeSH Terms]

**Embase Search strategy**

**#1** 'chronic otitis media'/exp/mj AND [article]/lim AND ([genetics]/lim OR [immunology and hematology]/lim OR [microbiology]/lim OR [otorhinolaryngology]/lim OR [pediatrics]/lim) AND [humans]/lim AND ([embase]/lim OR [embase classic]/lim)

**#2** 'chronic secretory otitis media'/exp/mj AND ([genetics]/lim OR [immunology and hematology]/lim OR [microbiology]/lim OR [otorhinolaryngology]/lim OR [pediatrics]/lim) AND [humans]/lim AND ([embase]/lim OR [embase classic]/lim)

**#3** 'chronic suppurative otitis media'/exp/mj AND ([genetics]/lim OR [immunology and hematology]/lim OR [microbiology]/lim OR [otorhinolaryngology]/lim OR [pediatrics]/lim) AND [humans]/lim AND ([embase]/lim OR [embase classic]/lim)

**#4** recurrent* AND 'otitis media'/exp/mj/dm_et,dm_ep,dm_cn,dm_pc AND [article]/lim AND ([anatomy and development]/lim OR [genetics]/lim OR [immunology and hematology]/lim OR [microbiology]/lim OR [otorhinolaryngology]/lim OR [pediatrics]/lim) AND [humans]/lim AND ([embase]/lim OR [embase classic]/lim)

**#5** #4 NOT 'acute otitis media'

**#6** 'cholesteatoma'/exp/mj/dm_et,dm_ep,dm_cn AND [article]/lim AND ([genetics]/lim OR [immunology and hematology]/lim OR [microbiology]/lim OR [otorhinolaryngology]/lim OR [pediatrics]/lim) AND [humans]/lim AND ([embase]/lim OR [embase classic]/lim)

**#7** #6 AND 'middle ear'

**#8** 'mastoiditis'/exp/mj/dm_et,dm_ep,dm_cn,dm_pc AND [article]/lim AND ([genetics]/lim OR [immunology and hematology]/lim OR [microbiology]/lim OR [otorhinolaryngology]/lim OR [pediatrics]/lim) AND [humans]/lim AND ([embase]/lim OR [embase classic]/lim)

**#9** #8 AND (chronic* OR recurrent*)

**#10** inflammat* AND 'middle ear'/exp/mj/dm_et,dm_ep,dm_cn,dm_pc AND [article]/lim AND ([genetics]/lim OR [immunology and hematology]/lim OR [microbiology]/lim OR [otorhinolaryngology]/lim OR [pediatrics]/lim) AND [humans]/lim AND ([embase]/lim OR [embase classic]/lim)

**#11** #1 OR #2 OR #3 OR #5 OR #7 OR #9 OR #10

**#12** #11 NOT 'case report'

CNKI search strategy

(((Otitis media) AND (chronic* OR recurrent*)) NOT “case report”)AND human

Wan Fang database

#1(Title: Chronic otitis media) AND Abstract: Epidemiology

#2 (Title: Chronic otitis media) AND Abstract: Etiology

#3 (Title: Recurrent otitis media) AND Abstract: Epidemiology

#4 (Title: Recurrent otitis media) AND Abstract: Etiology

#5 (Title: middle ear cholesteatoma) AND Abstract: Epidemiology

#6 (Title: middle ear cholesteatoma) AND Abstract: Etiology
